# Unilateral Alveolar Hemorrhage: A Rare Pulmonary Complication in Systemic Lupus Erythematosus

**DOI:** 10.7759/cureus.61491

**Published:** 2024-06-01

**Authors:** Jorge Nolasco-Amezcua, Hugo E González-Chávez, Rebecca Borrero-Colmenares, Karen S Arrazola-Mendoza, Francisco De la Peña-Camacho

**Affiliations:** 1 Department of Internal Medicine, General Hospital of the Institute of Security and Social Services of State Workers of Querétaro, Queretaro, MEX; 2 Department of Internal Medicine, Anahuac University Queretaro, Queretaro, MEX

**Keywords:** diffuse alveolar hemorrhage, lupus nephropathy, pulmonary disease, unilateral alveolar hemorraghe, sytemic lupus erythematosus

## Abstract

Systemic lupus erythematosus (SLE) is an autoimmune disease that presents a broad spectrum of clinical manifestations. Alveolar hemorrhage in SLE is rare and has a poor prognosis. We present the case of a patient with a diagnosis of SLE and lupus nephropathy on hemodialysis who presented criteria for alveolar hemorrhage with unilateral involvement, with clinical improvement after the administration of steroid boluses. The uncommon presentation of unilateral pulmonary involvement and the importance of making an adequate protocol for ruling out differential diagnoses are highlighted.

## Introduction

Systemic lupus erythematosus (SLE) is a complex disease considered the prototype of an autoimmune disease due to its antibody production and a wide range of clinical presentations. The more prevalent early manifestations are arthritis, photosensitive rash, cytopenias, and glomerulonephritis. When there is pulmonary involvement, the presentation comes later. Pleuropulmonary manifestations are classified as acute or chronic, ranging from subclinical pleural effusion to life-threatening alveolar hemorrhage [1]. Bilateral alveolar hemorrhage is a rare but critical manifestation, with a mortality rate of 50-90% and a prevalence of 0.5-5.7%; unilateral presentation is even rarer [2].

## Case presentation

A 27-year-old woman, diagnosed with SLE for 11 years, chronic kidney disease on hemodialysis secondary to class IV lupus nephropathy, and systemic arterial hypertension two years earlier, was admitted for a two-week period. Throughout her stay, she experienced a fever reaching up to 39 °C following hemodialysis sessions, along with myalgias and arthralgias. Paraclinical tests unveiled significant findings, including hyperchromic macrocytic anemia grade 3 (Hb 6.2 g/dL), severe thrombocytopenia (38,000 uL), leukocytes 2,950 uL with neutrophils 2,400 uL, urea 66.4 mg/dL, and creatinine 5.1 mg/dL.

During the course of the hospitalization, she experienced persistent episodes of fever with no additional symptoms. Empirical antibiotic therapy with vancomycin was started after cultures were taken due to suspicion of an infection at the vascular access site. However, subsequent negative cultures prompted the discontinuation of this treatment. Her Direct Coombs test returned positive with a score of +++, while her viral panel results for hepatitis A, B, C, and HIV were negative. Additionally, her torch profile was negative, and she exhibited low complement levels (C3 29.5 mg/dl, C4 3.81 mg/dl). Furthermore, her anti-Epstein-Barr antibodies and VDRL tests were nonreactive. Notably, her antinuclear antibodies were positive at a level of 3.3, while her antiphospholipid antibodies and antineutrophilic cytoplasmic antibodies were negative. Serial blood cultures and expectoration cultures yielded no evidence of microorganism growth. Echocardiography effectively ruled out the possibility of endocarditis.

**Figure 1:**
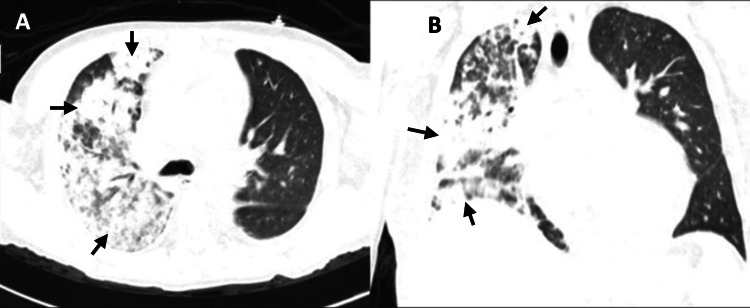
Chest CT lung window (A) Axial lung window. (B) Coronal reconstruction. Consolidation and diffuse centroacinar opacities were predominantly observed on the right lung during an acute unilateral alveolar hemorrhage event.

**Figure 2:**
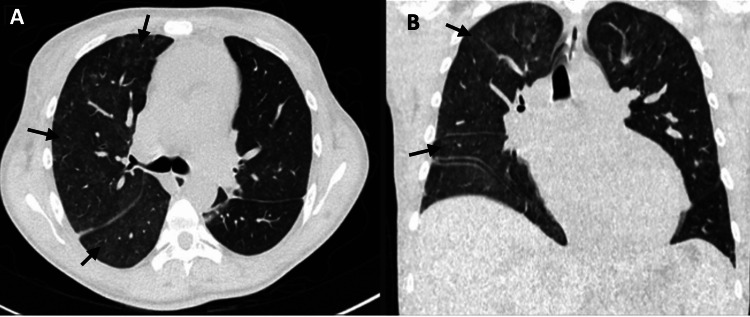
Chest CT lung window (A) Axial lung window. (B) Coronal reconstruction. Seventy-two hours after the initiation of immunosuppressive treatment, a few centroacinar ground-glass opacities of diffuse were observed.

One week after admission, the patient suddenly presented with dyspnea, pleuritic pain, hemoptysis, and tachypnea and required supplemental oxygen. Laboratory tests revealed a decrease in hemoglobin of more than 1 g/dl. Chest CT revealed consolidation and diffuse centroacinar opacities in the right lung parenchyma, indicative of alveolar hemorrhage (Figure 1). Methylprednisolone boluses (five doses) were administered, leading to clinical improvement and the resolution of fever. Three days later, a follow-up CT scan showed no evidence of lesions (Figure 2).

## Discussion

Alveolar hemorrhage is defined as the extravasation of red blood cells into the alveolar space, which is a consequence of injury or inflammation of the alveolar microcirculation and destruction of the alveolar-capillary basement membrane [3]. A correlation has been identified between diffuse alveolar hemorrhage and patients with SLE. The precise pathophysiology remains unclear, although it is postulated that immune activity plays a significant role. In patients with lupus nephritis who develop end-stage renal disease, approximately 80% are usually negative for activity markers. This is likely due to the immunodeficiency associated with the uremic state or dialysis, as well as the probable natural progression of the disease, which may result in the cessation of autoimmune activity. Consequently, pulmonary manifestations are uncommon in these patients [4].

This entity is strongly associated with disease activity, particularly in nephritis types III and IV (90%), elevated anti-dsDNA titers, low complement, a Systemic Lupus Erythematosus Disease Activity Index score >10, and neuropsychiatric manifestations [5]. The interval between the diagnosis of SLE and the onset of pulmonary involvement is variable, with an abrupt and transient clinical presentation (lasting less than seven days) that initially manifests as cough, hemoptysis (absent in 30-50% of cases), fever, and dyspnea [6]. In patients who present with hemoptysis, it is essential to rule out focal sources of pulmonary hemorrhage and sources of upper airway and gastrointestinal bleeding. Congestive heart failure, pneumonia, and other acute presentations of diffuse parenchymal lung disease must also be considered [3]. Microbiology studies are initially required to rule out infectious pathologies. The most common paraclinical findings are anemia, leukocytosis, and elevated acute phase reactants. However, these findings are not essential for the diagnosis of this entity. Since the pathology is hemorrhagic, it is imperative to rule out coagulation disorders [7]. Radiographically, new diffuse irregular opacities are observed, which, when corroborated by CT, are usually described as diffuse consolidations, without response to antibiotics and with resolution after initiation of high doses of systemic corticosteroids such as methylprednisolone 500-1,000 mg/day for up to five days, followed by gradual reduction. Approximately 80% of cases are described as bilateral alveolar-interstitial infiltrates; less than 20% are unilateral. Recurrent episodes of diffuse alveolar hemorrhage may culminate in pulmonary fibrosis or chronic reticular opacities. The usual method of diagnosis is by bronchoalveolar lavage, in which hemorrhagic effluent is found. Occasionally, when the diagnosis remains uncertain, a lung biopsy is required to corroborate or rule out the diagnosis [8,9].

Concurrent pneumonia in patients with diffuse alveolar hemorrhage and SLE is common (32-45%); thus, empiric antibiotic coverage is recommended before the onset of immunosuppression. The most frequently reported microorganisms are *Pseudomonas* spp., *Aspergillus*, *Staphylococcus aureus*, and cytomegalovirus [10].

The mortality rate has been reported to be as high as 90%. However, in the last 15 years, it has ranged between 40% and 60%. Factors associated with higher mortality include the presence of concomitant pulmonary infection, renal failure, the need for mechanical ventilation, thrombocytopenia, and a high Acute Physiology and Chronic Health Evaluation II (APACHE II) score. Of these, the last four are the most frequently associated factors [5].

## Conclusions

The case of unilateral alveolar hemorrhage in an SLE patient described above is unusual due to its clinical presentation; it was necessary to rule out differential diagnoses of hemoptysis and fever. The reduction in hemoglobin levels and the presence of hemoptysis were key factors in the diagnosis of this condition. A chest CT revealed an unusual pattern of centroacinar opacities with a unilateral distribution. When steroid pulses were initiated, the tomographic pattern exhibited an immediate improvement, which is consistent with the findings described in the literature. Given the limitations of the hospital and the evidence of clinical improvement and tomographic resolution, it was determined that bronchoscopy and bronchoalveolar lavage would not be performed. Early recognition of this rare condition allows the establishment of adequate and timely treatment, reducing sequelae, complications, and even death in such serious situations.
